# Editorial: Stem cell exhaustion in aging

**DOI:** 10.3389/fragi.2024.1433702

**Published:** 2024-05-31

**Authors:** Sarallah Rezazadeh, Georgina May Ellison-Hughes

**Affiliations:** ^1^ Department of Neurology, Icahn School of Medicine at Mount Sinai, New York, NY, United States; ^2^ School of Basic and Medical Biosciences, Faculty of Life Sciences & Medicine, King’s College London, London, England, United Kingdom

**Keywords:** quiescence, senscence, hair follicle stem cell, atac seq, oocyte, neural stem cell (NSC), exosome

This Research Topic aimed to gather research works providing insights into the mechanisms of stem cell exhaustion in aging. Our understanding of the role of stem cell homeostasis has expanded rapidly over the past few years and provided new avenues for treatment, and prevention of age-related disorders. Therefore, it is critical to understand the role of stem cell turnover in certain tissues where stem cells could be isolated in abundance to comprehend the impact of molecular regulation of stem cell homeostasis in aging and use that knowledge for cell-based therapies.

The excellent studies published in this Research Topic [references (Zhang et al.; Micheli et al.; Nagamatsu; Zhang et al.)] will allow us to highlight and address some of the gaps in knowledge mentioned earlier.

SCs in aging tissues have limited functional output, which inevitably leads to fitness decline of tissue (Lopez-Otin et al., 2023). What accounts for this age-related decline in SC activity remains unclear. However, recent studies suggest the balance between proliferation *versus* quiescence is fundamental to tissue SCs maintenance over the course of a lifetime of repeated environmental insults (Cheung and Rando, 2013). Quiescence, through restricting the number of SC divisions is known to play an important role in long term SC maintenance, and loss of quiescence often results in an imbalance in progenitor cell populations ultimately leading to stem cell depletion. As a result, tissue replenishment is affected during homeostasis and after damage (Brack and Rando, 2012).

Over the last decade, advances in purifying quiescent stem cells have allowed the use of high-throughput techniques such as single cell RNA-sequencing, single-cell Assay for Transposase-Accessible Chromatin (ATAC) sequencing, and chromatin immunoprecipitation followed by sequencing (ChIP-Seq). In tissue compartments such as skin and muscle, where such techniques could be used because of stem cell abundance, these approaches have provided important key information regarding the transcriptional nature of quiescence (Cheung and Rando, 2013). For instance, in a hallmark study it was shown that only few genes are marked by bivalent domains in lineage-restricted, quiescent hair follicle stem cells (HF-SCs) (Adam and Fuchs, 2016). Intriguingly, in quiescent HF-SCs thousands of genes are marked by the H3K4me3 mark (which is associated with active transcription), despite low transcriptional profile of these cells (Adam and Fuchs, 2016). We know that epigenetics play role in governing HF-SC fate, but the mechanism behind this transcriptional control is yet to be fully understood.

In line with above studies, Zhang paper (Zhang et al.) discusses alterations of the open chromatin landscape in different HF-SC states. Through single cell analysis of open chromatin landscape by ATAC sequencing in HF-SC obtained from young, middle-age and old mice, Zhang et al. showed reduced open chromatin regions is associated with differentiation whereas enhanced open chromatin regions in HF-SCs are associated with quiescence. This study also identified bifurcating trajectories of HF-SC state and provided evidence that HF-SC and niche cells age differently. In particular, HF-SC can maintain their identity over time, but niche cells undergo more profound changes during the course of aging.

In a further research article (Zhang et al.) highlighted the importance of exosomes in transition into the quiescent state. Using proteomics approaches they identified key factors important for the maintenance of the quiescent state, amongst which proteins involved in translation machinery, and specifically 40S subunit components, were significantly enriched in exosomes obtained from quiescent cells. Zhang et al. showed that upon inhibition of exosome, the transition into quiescence state was delayed, and instead quiescent neural stem cells (NSCs) exited from the G0 phase to initiate the cell cycle through upregulation of *CyclinD1*. This data is consistent with the “discarding model” of exosome function which states exosomes exhaust the functional translational machinery of quiescent NSCs to reduce new protein synthesis for proliferation. The remaining question is how during aging the kinetics of exosome production could contribute to the balance between staying dormant and entering an active state of stemness.

It has been proposed that decrease in tissue stem cells is at least partially caused by the senescence of progenitors with age (Campisi, 2005). These declines in progenitor frequency and function correlate with increased expression of p16^INK4a^, which encodes a cyclin-dependent kinase inhibitor linked to senescence (Campisi, 2005; Molofsky et al., 2006). Interestingly, ageing p16^INK4a^-deficient mice showed less decline in subventricular zone proliferation, olfactory bulb neurogenesis. (Molofsky et al., 2006). Surprisingly, p16^INK4a^ deficiency did not significantly impact progenitor function in the dentate gyrus or enteric nervous system, suggesting regional differences in the response of neural progenitors to increased p16^INK4a^ expression during ageing (Molofsky et al., 2006). In line with the original observation about the role of p16^INK4a^ *in adult neurogenesis, the recent paper by* Micheli et al. *showed* that p16Ink4a ablation by itself does not activate stem/progenitor cells subgranular zone of dentate gyrus. However, exercise strongly induced stem cell proliferation in p16^INK4a^ knockout dentate gyrus, but not in wild type (Micheli et al.). To reach to this conclusion, authors performed five pairwise comparisons of two genotypes (p16^INK4a^ KO and p16^INK4a^ WT) vs. two treatments (RUN and CTL) relative to the expression of genes in the dentate gyrus, as well as the intersection of the differentially expressed genes in these 5 sets. They found a set of 106 genes which expression specifically reflects the pattern of proliferative response of p16^INK4a^ knockout stem cells to running exercise. These genes are involved in processes that regulate stem cell activation, such as synaptic function, neurotransmitter metabolism, stem cell proliferation control, and reactive oxygen species level regulation. The million-dollar question was whether a second set of exercise would trigger the same neurogenesis pattern in the absence of p16^INK4a^. Towards this, p16 wild-type and knockout mice were subjected to two consecutive voluntary running sessions, spaced 3 weeks apart. Intriguingly, the second running exercise did not induce a proliferative response in p16 knockout stem cells. This suggests that the process of stem cell activation is strictly regulated and/or undergoes exhaustion.

Among the main regulators of adult NSC quiescence, a central role is exerted by the Cip/Kip family of Cyclin dependent kinase inhibitors (CKIs), which comprises p21^Waf1/Cip1^, p27^Kip1^, and p57^Kip2^ (Coqueret, 2003). Among which p21 is a key mediator of the p53-induced G1 checkpoint via the binding to and inactivation of G1-associated cyclin A–and cyclin E–containing cyclin/CDK complexes (Karimian et al., 2016). In p21 KO mice, a rapid activation of quiescent NSCs in the post-natal stage has been reported (Farioli-Vecchioli and Tirone, 2015). Like p16 KO, running exercise in p21 knockdown massively increases neurogenesis in the subventricular zone (SVZ) of p21 KO mice (Nicolis di Robilant et al., 2019). These observations put cell cycle regulators, such as p21 and p16 at the heart of NSC turnover. However, further investigations are needed to reveal a clearer picture of the molecular mechanisms underpinning the enduring self-renewal capacity of p21 and p16-KO NSCs, with the ultimate goal of exploiting them as a potential source of active NSCs able to repair brain damage or counteract neurodegenerative processes (Nicolis di Robilant et al., 2019).

Perhaps, ovary is one of the earliest organs where function declines in aging. In ovary, oocytes enter meiosis at embryonic stage, where they maintain sustainable oogenesis without stem cells (Micheli et al.). In a review article Nagamatsu summarizes the newest findings in aging oocyte and the genes responsible for the maintenance of the quiescence state. The author discusses similarities between oocytes and hematopoietic stem cells (HSC). Oocytes and HSCs prefer glycolysis over oxidative phosphorylation, and both seem to be maintained under hypoxic conditions in their microenvironment (Ito and Suda, 2014). This puts mitochondrial metabolism at the heart of both oocyte physiology and HSC aging. Recent reports show that defective mitochondrial function increases reactive oxygen species (ROS) levels in aged oocytes. Given the contribution of ROS to macromolecular damage, excessive ROS which is generated through mitochondrial malfunction can contribute to oocyte aging.

One way to reduce the metabolic burden was shown to be caloric restriction. The beneficial impact of caloric restriction in multiple stem cell types, such as intestinal, muscle, HSCs, and NSCs has been reported previously. Consistent with above, caloric restriction also prevents functional decline in oocytes at multiple levels (Mishina et al., 2021). Intriguingly, a downstream effector of caloric restriction is NAD^+^ pool and sirtuins (SIRT) gene family. In particular, SIRT1 activation was suggested to be the mechanism behind Resveratrol-mediated prevention of oocyte aging (Grzeczka and Kordowitzki, 2022). While all these findings suggest intriguing clinical interventions, the path towards saving oocytes from aging seems to not be straightforward. The most challenging Research Topic is the long-term *in vitro* culture system of oocytes. Besides, lack of reliable oocyte quiescence markers makes distinguishing active and quiescent oocytes technically unfeasible. Moving forward, improvement of the *in vitro* systems would pave the way for much improved understanding of the aging of primordial follicles and oocyte growth.

In summary, the studies published in this Research Topic demonstrate how far our understanding of the molecular mechanisms of stem cell quiescence has come in the last 5 years. However, these studies also highlight the work that needs to be done to improve our understanding of adult stem cell homeostasis. Nevertheless, adult stem cell quiescence and senescence represent a major mechanistic basis, predictive biomarkers, and therapeutic target of various age-associated complex diseases.

## References

[B1] Adam R. C. Fuchs E. (2016). The yin and yang of chromatin dynamics in stem cell fate selection. Trends Genet. 32 (2), 89–100. 10.1016/j.tig.2015.11.002 26689127 PMC4733603

[B2] Brack A. S. Rando T. A. (2012). Tissue-specific stem cells: lessons from the skeletal muscle satellite cell. Cell. Stem Cell. 10 (5), 504–514. 10.1016/j.stem.2012.04.001 22560074 PMC3348769

[B3] Campisi J. (2005). Senescent cells, tumor suppression, and organismal aging: good citizens, bad neighbors. Cell. 120 (4), 513–522. 10.1016/j.cell.2005.02.003 15734683

[B4] Cheung T. H. Rando T. A. (2013). Molecular regulation of stem cell quiescence. Nat. Rev. Mol. Cell. Biol. 14 (6), 329–340. 10.1038/nrm3591 23698583 PMC3808888

[B5] Coqueret O. (2003). New roles for p21 and p27 cell-cycle inhibitors: a function for each cell compartment? Trends Cell. Biol. 13 (2), 65–70. 10.1016/s0962-8924(02)00043-0 12559756

[B6] Farioli-Vecchioli S. Tirone F. (2015). Control of the cell cycle in adult neurogenesis and its relation with physical exercise. Brain Plast. 1 (1), 41–54. 10.3233/BPL-150013 29765834 PMC5928538

[B7] Grzeczka A. Kordowitzki P. (2022). Resveratrol and SIRT1: antiaging cornerstones for oocytes? Nutrients 14 (23), 5101. 10.3390/nu14235101 36501130 PMC9736670

[B8] Ito K. Suda T. (2014). Metabolic requirements for the maintenance of self-renewing stem cells. Nat. Rev. Mol. Cell. Biol. 15 (4), 243–256. 10.1038/nrm3772 24651542 PMC4095859

[B9] Karimian A. Ahmadi Y. Yousefi B. (2016). Multiple functions of p21 in cell cycle, apoptosis and transcriptional regulation after DNA damage. DNA Repair (Amst). 42, 63–71. 10.1016/j.dnarep.2016.04.008 27156098

[B10] Lopez-Otin C. Blasco M. A. Partridge L. Serrano M. Kroemer G. (2023). Hallmarks of aging: an expanding universe. Cell. 186 (2), 243–278. 10.1016/j.cell.2022.11.001 36599349

[B11] Mishina T. Tabata N. Hayashi T. Yoshimura M. Umeda M. Mori M. (2021). Single-oocyte transcriptome analysis reveals aging-associated effects influenced by life stage and calorie restriction. Aging Cell. 20 (8), e13428. 10.1111/acel.13428 34245092 PMC8373347

[B12] Molofsky A. V. Slutsky S. G. Joseph N. M. He S. Pardal R. Krishnamurthy J. (2006). Increasing p16INK4a expression decreases forebrain progenitors and neurogenesis during ageing. Nature 443 (7110), 448–452. 10.1038/nature05091 16957738 PMC2586960

[B13] Nicolis di Robilant V. Scardigli R. Strimpakos G. Tirone F. Middei S. Scopa C. (2019). Running-activated neural stem cells enhance subventricular neurogenesis and improve olfactory behavior in p21 knockout mice. Mol. Neurobiol. 56 (11), 7534–7556. 10.1007/s12035-019-1590-6 31062248

